# {2-[2-(Ethyl­amino)ethyl­imino­meth­yl]-5-methoxy­phenolato}thio­cyanato­nickel(II)

**DOI:** 10.1107/S1600536810002357

**Published:** 2010-01-23

**Authors:** Lin Liu

**Affiliations:** a College of Chemistry and Biology Engineering, Yichun University, Yichun 336000, People’s Republic of China

## Abstract

In the title mononuclear nickel(II) complex, [Ni(C_12_H_17_N_2_O_2_)(NCS)], the metal atom is four-coordinated in a tetra­hedrally distorted square-planar geometry by the phenolate O atom, the imine N atom and the amine N atom of the Schiff base ligand and by the N atom of a thio­cyanate ligand. In the crystal structure, centrosymmetrically related mol­ecules are linked into dimers through inter­molecular N—H⋯O hydrogen bonds. These dimers are further connected by inter­molecular C—H⋯S hydrogen bonds, forming chains running parallel to [101].

## Related literature

For general background to nickel(II) complexes with Schiff bases, see: Campbell & Urbach (1973 ▶); Wallis & Cummings (1974 ▶); Polt *et al.* (2003 ▶); Mukhopadhyay *et al.* (2003 ▶). For the structures of related complexes, see: Montazerozohori *et al.* (2009 ▶); Zhu *et al.* (2004 ▶, 2006 ▶). 
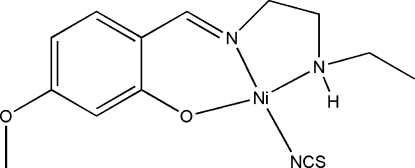


## Experimental

### 

#### Crystal data

[Ni(C_12_H_17_N_2_O_2_)(NCS)]
*M* *_r_* = 338.07
Monoclinic, 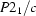

*a* = 9.298 (7) Å
*b* = 19.679 (14) Å
*c* = 8.461 (7) Å
β = 111.716 (11)°
*V* = 1438.3 (19) Å^3^
*Z* = 4
Mo *K*α radiation
μ = 1.50 mm^−1^
*T* = 298 K
0.23 × 0.20 × 0.20 mm

#### Data collection

Bruker SMART CCD area-detector diffractometer
Absorption correction: multi-scan (*SADABS*; Bruker, 1998 ▶) *T*_min_ = 0.725, *T*_max_ = 0.754
6520 measured reflections
2500 independent reflections
1564 reflections with *I* > 2σ(*I*)
*R*_int_ = 0.071

#### Refinement

*R*[*F* ^2^ > 2σ(*F* ^2^)] = 0.052
*wR*(*F* ^2^) = 0.127
*S* = 1.08
2500 reflections
186 parameters
1 restraint
H atoms treated by a mixture of independent and constrained refinement
Δρ_max_ = 0.40 e Å^−3^
Δρ_min_ = −0.52 e Å^−3^

### 

Data collection: *SMART* (Bruker, 1998 ▶); cell refinement: *SAINT* (Bruker, 1998 ▶); data reduction: *SAINT*; program(s) used to solve structure: *SHELXS97* (Sheldrick, 2008 ▶); program(s) used to refine structure: *SHELXL97* (Sheldrick, 2008 ▶); molecular graphics: *SHELXTL* (Sheldrick, 2008 ▶); software used to prepare material for publication: *SHELXTL*.

## Supplementary Material

Crystal structure: contains datablocks global, I. DOI: 10.1107/S1600536810002357/rz2410sup1.cif

Structure factors: contains datablocks I. DOI: 10.1107/S1600536810002357/rz2410Isup2.hkl

Additional supplementary materials:  crystallographic information; 3D view; checkCIF report

## Figures and Tables

**Table 1:** Hydrogen-bond geometry (Å, °)

*D*—H⋯*A*	*D*—H	H⋯*A*	*D*⋯*A*	*D*—H⋯*A*
N2—H2⋯O1^i^	0.90 (5)	2.25 (3)	3.059 (6)	150 (5)
C7—H7⋯S1^ii^	0.93	2.83	3.708 (6)	158

Symmetry codes: (i) 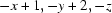; (ii) 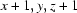.

**Fig. 1.**
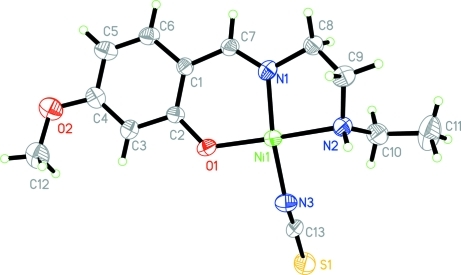
The molecular structure of the title complex, with 30% displacement ellipsoids.

**Fig. 2.**
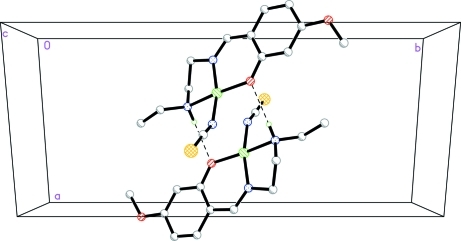
Partial crystal packing of the title compound showing the formation of a dimer through N—H···O hydrogen bonds. Hydrogen atoms not involved in hydrogen interactions are omitted for clarity.

## supplementary crystallographic information

### Comment

Nickel(II) complexes with Schiff bases have been extensively studied (Campbell & Urbach, 1973; Wallis & Cummings, 1974; Polt *et al.*, 2003; Mukhopadhyay *et al.*, 2003). In the title compound, the Ni atom is four-coordinate by the phenolate O atom, imine N atom, and amine N atom of the Schiff base ligand, and by the N atom of a thiocyanate ligand, forming a tetrahedrally distorted square-planar geometry (Fig. 1). Bond lengths and angles involving the metal atom are comparable with those observed in similar complexes (Montazerozohori *et al.*, 2009; Zhu *et al.*, 2004; Zhu *et al.*, 2006). The Ni1/N1/O1/C1/C2/C7 six-membered chelate ring is approximately planar (maximum deviation 0.063 (4) Å for atom N1, the Ni1/N1/N2/C8/C8 five-membered chelate ring assumes an envelope conformation, with atom C9 displaced by 0.589 (6) Å from the mean plane of the other atoms. In the crystal structure, centrosymmetrically related complex molecules are linked through intermolecular N—H···O hydrogen bonds (Table 1), forming a dimer (Fig. 2). The dimers are further connected by C—H···S hydrogen bonds, forming chains running parallel to [101].

### Experimental

Equimolar quantities (0.1 mmol each) of *N*-ethylethane-1,2-diamine, ammonium thiocyanate, and Ni(CH_3_COO)_2_.4H_2_O were mixed and stirred in a methanol solution for 30 min at reflux. After keeping the filtrate in air for a few days, red block crystals were formed on slow evaporation of the solvent.

### Refinement

Atom H2 was located in a difference Fourier map and refined isotropically, with the N—H distance restrained to 0.90 (1) Å. Other H atoms were placed in calculated positions and constrained to ride on their parent atoms, with C—H distances in the range 0.93–0.97 Å, and with *U*_iso_(H) = 1.2*U*_eq_(C) or 1.5*U*_eq_(C) for methyl H atoms.

### Crystal data

**Table:**

[Ni(C_12_H_17_N_2_O_2_)(NCS)]	*F*(000) = 704
*M**_r_* = 338.07	*D*_x_ = 1.561 Mg m^−^^3^
Monoclinic, *P*2_1_/*c*	Mo *K*α radiation, λ = 0.71073 Å
Hall symbol: -P 2ybc	Cell parameters from 1958 reflections
*a* = 9.298 (7) Å	θ = 2.4–24.5°
*b* = 19.679 (14) Å	µ = 1.50 mm^−^^1^
*c* = 8.461 (7) Å	*T* = 298 K
β = 111.716 (11)°	Block, red
*V* = 1438.3 (19) Å^3^	0.23 × 0.20 × 0.20 mm
*Z* = 4	

### Data collection

**Table:**

Bruker SMART CCD area-detector diffractometer	2500 independent reflections
Radiation source: fine-focus sealed tube	1564 reflections with *I* > 2σ(*I*)
graphite	*R*_int_ = 0.071
ω scan	θ_max_ = 25.1°, θ_min_ = 2.4°
Absorption correction: multi-scan (*SADABS*; Sheldrick, 2008)	*h* = −10→11
*T*_min_ = 0.725, *T*_max_ = 0.754	*k* = −23→16
6520 measured reflections	*l* = −9→9

### Refinement

**Table:**

Refinement on *F*^2^	Primary atom site location: structure-invariant direct methods
Least-squares matrix: full	Secondary atom site location: difference Fourier map
*R*[*F*^2^ > 2σ(*F*^2^)] = 0.052	Hydrogen site location: inferred from neighbouring sites
*wR*(*F*^2^) = 0.127	H atoms treated by a mixture of independent and constrained refinement
*S* = 1.08	*w* = 1/[σ^2^(*F*_o_^2^) + (0.0494*P*)^2^ + 0.2075*P*] where *P* = (*F*_o_^2^ + 2*F*_c_^2^)/3
2500 reflections	(Δ/σ)_max_ = 0.001
186 parameters	Δρ_max_ = 0.40 e Å^−^^3^
1 restraint	Δρ_min_ = −0.52 e Å^−^^3^

### Special details

**Table:**

Geometry. All e.s.d.'s (except the e.s.d. in the dihedral angle between two l.s. planes) are estimated using the full covariance matrix. The cell e.s.d.'s are taken into account individually in the estimation of e.s.d.'s in distances, angles and torsion angles; correlations between e.s.d.'s in cell parameters are only used when they are defined by crystal symmetry. An approximate (isotropic) treatment of cell e.s.d.'s is used for estimating e.s.d.'s involving l.s. planes.
Refinement. Refinement of *F*^2^ against ALL reflections. The weighted *R*-factor *wR* and goodness of fit *S* are based on *F*^2^, conventional *R*-factors *R* are based on *F*, with *F* set to zero for negative *F*^2^. The threshold expression of *F*^2^ > σ(*F*^2^) is used only for calculating *R*-factors(gt) *etc*. and is not relevant to the choice of reflections for refinement. *R*-factors based on *F*^2^ are statistically about twice as large as those based on *F*, and *R*- factors based on ALL data will be even larger.

### Fractional atomic coordinates and isotropic or equivalent isotropic displacement parameters (Å^2^)

**Table:**

	*x*	*y*	*z*	*U*_iso_*/*U*_eq_	
Ni1	0.66858 (7)	0.96690 (3)	0.04177 (8)	0.0404 (2)	
N1	0.8603 (5)	0.9561 (2)	0.2264 (5)	0.0469 (11)	
N2	0.5948 (5)	0.8919 (2)	0.1568 (6)	0.0493 (11)	
N3	0.4937 (5)	0.9566 (2)	−0.1713 (6)	0.0522 (12)	
O1	0.7312 (4)	1.04775 (15)	−0.0367 (4)	0.0453 (9)	
O2	1.0527 (5)	1.2299 (2)	−0.0529 (5)	0.0685 (12)	
S1	0.3559 (2)	0.90996 (10)	−0.4968 (2)	0.1057 (8)	
C1	0.9916 (6)	1.0501 (2)	0.1607 (6)	0.0430 (13)	
C2	0.8670 (6)	1.0760 (3)	0.0252 (6)	0.0401 (12)	
C3	0.8866 (6)	1.1367 (2)	−0.0506 (6)	0.0459 (13)	
H3	0.8042	1.1550	−0.1406	0.055*	
C4	1.0268 (7)	1.1694 (3)	0.0070 (7)	0.0501 (14)	
C5	1.1530 (7)	1.1415 (3)	0.1362 (7)	0.0557 (15)	
H5	1.2489	1.1629	0.1717	0.067*	
C6	1.1350 (6)	1.0835 (3)	0.2092 (7)	0.0516 (14)	
H6	1.2202	1.0646	0.2947	0.062*	
C7	0.9819 (7)	0.9921 (3)	0.2524 (6)	0.0475 (13)	
H7	1.0719	0.9784	0.3402	0.057*	
C8	0.8622 (6)	0.8990 (2)	0.3367 (6)	0.0534 (15)	
H8A	0.9347	0.9074	0.4515	0.064*	
H8B	0.8929	0.8577	0.2949	0.064*	
C9	0.7018 (6)	0.8923 (3)	0.3346 (7)	0.0562 (15)	
H9A	0.6919	0.8504	0.3903	0.067*	
H9B	0.6784	0.9300	0.3947	0.067*	
C10	0.5750 (7)	0.8253 (3)	0.0735 (8)	0.0659 (17)	
H10A	0.6760	0.8084	0.0835	0.079*	
H10B	0.5144	0.8312	−0.0467	0.079*	
C11	0.4994 (8)	0.7737 (3)	0.1418 (11)	0.112 (3)	
H11A	0.5622	0.7648	0.2586	0.168*	
H11B	0.4869	0.7326	0.0770	0.168*	
H11C	0.3998	0.7900	0.1342	0.168*	
C12	0.9247 (8)	1.2632 (3)	−0.1721 (8)	0.080 (2)	
H12A	0.8835	1.2361	−0.2733	0.120*	
H12B	0.9566	1.3065	−0.1999	0.120*	
H12C	0.8466	1.2698	−0.1247	0.120*	
C13	0.4377 (6)	0.9358 (3)	−0.3067 (8)	0.0506 (14)	
H2	0.507 (4)	0.908 (3)	0.164 (7)	0.080*	

### Atomic displacement parameters (Å^2^)

**Table:**

	*U*^11^	*U*^22^	*U*^33^	*U*^12^	*U*^13^	*U*^23^
Ni1	0.0370 (4)	0.0427 (4)	0.0389 (4)	−0.0023 (3)	0.0108 (3)	0.0030 (3)
N1	0.046 (3)	0.051 (3)	0.046 (3)	0.007 (2)	0.021 (2)	0.009 (2)
N2	0.045 (3)	0.049 (3)	0.052 (3)	0.002 (2)	0.016 (2)	0.001 (2)
N3	0.046 (3)	0.061 (3)	0.048 (3)	−0.009 (2)	0.017 (2)	0.004 (2)
O1	0.038 (2)	0.049 (2)	0.045 (2)	0.0006 (17)	0.0100 (17)	0.0081 (15)
O2	0.066 (3)	0.070 (3)	0.077 (3)	−0.021 (2)	0.036 (3)	0.003 (2)
S1	0.1060 (17)	0.1090 (16)	0.0647 (12)	0.0371 (12)	−0.0118 (11)	−0.0307 (10)
C1	0.047 (4)	0.047 (3)	0.037 (3)	0.005 (3)	0.018 (3)	−0.004 (2)
C2	0.033 (3)	0.052 (3)	0.039 (3)	−0.002 (3)	0.018 (3)	−0.007 (2)
C3	0.045 (3)	0.050 (3)	0.049 (3)	0.002 (3)	0.024 (3)	0.004 (2)
C4	0.058 (4)	0.054 (3)	0.051 (4)	−0.005 (3)	0.036 (3)	−0.001 (3)
C5	0.043 (4)	0.077 (4)	0.056 (4)	−0.015 (3)	0.028 (3)	−0.008 (3)
C6	0.037 (3)	0.071 (4)	0.050 (3)	0.001 (3)	0.019 (3)	−0.006 (3)
C7	0.040 (3)	0.057 (3)	0.045 (3)	0.007 (3)	0.014 (3)	−0.005 (3)
C8	0.059 (4)	0.048 (3)	0.046 (3)	0.004 (3)	0.011 (3)	0.007 (2)
C9	0.057 (4)	0.064 (4)	0.049 (4)	0.001 (3)	0.020 (3)	0.011 (3)
C10	0.067 (4)	0.053 (4)	0.076 (4)	−0.010 (3)	0.024 (4)	−0.001 (3)
C11	0.099 (6)	0.053 (4)	0.213 (9)	−0.003 (4)	0.092 (6)	0.008 (5)
C12	0.095 (5)	0.067 (4)	0.087 (5)	−0.015 (4)	0.044 (5)	0.019 (4)
C13	0.043 (4)	0.050 (3)	0.053 (4)	0.011 (3)	0.011 (3)	0.002 (3)

### Geometric parameters (Å, °)

**Table:**

Ni1—O1	1.897 (3)	C4—C5	1.387 (7)
Ni1—N1	1.898 (4)	C5—C6	1.338 (7)
Ni1—N3	1.940 (5)	C5—H5	0.9300
Ni1—N2	2.023 (5)	C6—H6	0.9300
N1—C7	1.281 (6)	C7—H7	0.9300
N1—C8	1.457 (6)	C8—C9	1.491 (7)
N2—C9	1.465 (6)	C8—H8A	0.9700
N2—C10	1.467 (7)	C8—H8B	0.9700
N2—H2	0.90 (5)	C9—H9A	0.9700
N3—C13	1.144 (6)	C9—H9B	0.9700
O1—C2	1.299 (5)	C10—C11	1.469 (8)
O2—C4	1.351 (6)	C10—H10A	0.9700
O2—C12	1.405 (7)	C10—H10B	0.9700
S1—C13	1.588 (6)	C11—H11A	0.9600
C1—C2	1.391 (7)	C11—H11B	0.9600
C1—C7	1.403 (7)	C11—H11C	0.9600
C1—C6	1.404 (7)	C12—H12A	0.9600
C2—C3	1.399 (7)	C12—H12B	0.9600
C3—C4	1.371 (7)	C12—H12C	0.9600
C3—H3	0.9300		
			
O1—Ni1—N1	93.75 (17)	C1—C6—H6	119.0
O1—Ni1—N3	91.31 (16)	N1—C7—C1	126.0 (5)
N1—Ni1—N3	164.13 (18)	N1—C7—H7	117.0
O1—Ni1—N2	169.56 (16)	C1—C7—H7	117.0
N1—Ni1—N2	84.54 (18)	N1—C8—C9	106.6 (4)
N3—Ni1—N2	93.08 (18)	N1—C8—H8A	110.4
C7—N1—C8	121.0 (5)	C9—C8—H8A	110.4
C7—N1—Ni1	125.4 (4)	N1—C8—H8B	110.4
C8—N1—Ni1	113.5 (3)	C9—C8—H8B	110.4
C9—N2—C10	114.5 (4)	H8A—C8—H8B	108.6
C9—N2—Ni1	105.5 (3)	N2—C9—C8	108.0 (4)
C10—N2—Ni1	115.2 (4)	N2—C9—H9A	110.1
C9—N2—H2	103 (4)	C8—C9—H9A	110.1
C10—N2—H2	113 (4)	N2—C9—H9B	110.1
Ni1—N2—H2	105 (4)	C8—C9—H9B	110.1
C13—N3—Ni1	151.9 (5)	H9A—C9—H9B	108.4
C2—O1—Ni1	126.6 (3)	N2—C10—C11	114.9 (6)
C4—O2—C12	117.3 (5)	N2—C10—H10A	108.5
C2—C1—C7	123.4 (5)	C11—C10—H10A	108.5
C2—C1—C6	118.8 (5)	N2—C10—H10B	108.5
C7—C1—C6	117.9 (5)	C11—C10—H10B	108.5
O1—C2—C1	124.4 (5)	H10A—C10—H10B	107.5
O1—C2—C3	116.9 (5)	C10—C11—H11A	109.5
C1—C2—C3	118.7 (5)	C10—C11—H11B	109.5
C4—C3—C2	120.4 (5)	H11A—C11—H11B	109.5
C4—C3—H3	119.8	C10—C11—H11C	109.5
C2—C3—H3	119.8	H11A—C11—H11C	109.5
O2—C4—C3	123.9 (5)	H11B—C11—H11C	109.5
O2—C4—C5	115.4 (5)	O2—C12—H12A	109.5
C3—C4—C5	120.7 (5)	O2—C12—H12B	109.5
C6—C5—C4	119.2 (5)	H12A—C12—H12B	109.5
C6—C5—H5	120.4	O2—C12—H12C	109.5
C4—C5—H5	120.4	H12A—C12—H12C	109.5
C5—C6—C1	122.1 (5)	H12B—C12—H12C	109.5
C5—C6—H6	119.0	N3—C13—S1	177.6 (5)

### Hydrogen-bond geometry (Å, °)

**Table:**

*D*—H···*A*	*D*—H	H···*A*	*D*···*A*	*D*—H···*A*
N2—H2···O1^i^	0.90 (5)	2.25 (3)	3.059 (6)	150 (5)
C7—H7···S1^ii^	0.93	2.83	3.708 (6)	158

Symmetry codes: (i) −*x*+1, −*y*+2, −*z*; (ii) *x*+1, *y*, *z*+1.

## References

[bb1] Bruker (1998). *SADABS*, *SMART* and *SAINT* Bruker AXS Inc., Madison, Wisconsin, USA.

[bb2] Campbell, T. B. & Urbach, F. L. (1973). *Inorg. Chem.* **12**, 1840–1846.

[bb3] Montazerozohori, M., Habibi, M. H., Mokhtari, R., Yamane, Y. & Suzuki, T. (2009). *Acta Cryst.* E**65**, m703. 10.1107/S1600536809019965 PMC2969458 21582649

[bb4] Mukhopadhyay, S., Mandal, D., Ghosh, D., Goldberg, I. & Chaudhury, M. (2003). *Inorg. Chem.* **42**, 8439–8445. 10.1021/ic0346174 14658897

[bb5] Polt, R., Kelly, B. D., Dangel, B. D., Tadikonda, U. B., Ross, R. E., Raitsimring, A. M. & Astashkin, A. V. (2003). *Inorg. Chem.* **42**, 566–574. 10.1021/ic025996o 12693240

[bb6] Sheldrick, G. M. (2008). *Acta Cryst.* A**64**, 112–122. 10.1107/S0108767307043930 18156677

[bb7] Wallis, W. N. & Cummings, S. C. (1974). *Inorg. Chem.* **13**, 991–994.

[bb8] Zhu, B., Ruang, W. & Zhu, Z. (2004). *Acta Cryst.* E**60**, m634–m636.

[bb9] Zhu, C.-G., Wang, F.-W. & Wei, Y.-J. (2006). *Acta Cryst.* E**62**, m1816–m1817.

